# Antipsychotic Drug Responsiveness and Dopamine Receptor Signaling; Old Players and New Prospects

**DOI:** 10.3389/fpsyt.2018.00702

**Published:** 2019-01-09

**Authors:** Antonio Rampino, Aleksandra Marakhovskaia, Tiago Soares-Silva, Silvia Torretta, Federica Veneziani, Jean Martin Beaulieu

**Affiliations:** ^1^Department of Basic Medical Science, Neuroscience and Sense Organs, University of Bari “Aldo Moro”, Bari, Italy; ^2^Azienda Ospedaliero-Universitaria Consorziale Policlinico di Bari, Bari, Italy; ^3^Department of Pharmacology and Toxicology, University of Toronto, Toronto, ON, Canada

**Keywords:** dopamine, risk factors, antipsychotic agents, genetic variants, schizophrenia therapy

## Abstract

Antipsychotic drugs targeting dopamine neurotransmission are still the principal mean of therapeutic intervention for schizophrenia. However, about one third of people do not respond to dopaminergic antipsychotics. Genome wide association studies (GWAS), have shown that multiple genetic factors play a role in schizophrenia pathophysiology. Most of these schizophrenia risk variants are not related to dopamine or antipsychotic drugs mechanism of action. Genetic factors have also been implicated in defining response to antipsychotic medication. In contrast to disease risk, variation of genes coding for molecular targets of antipsychotics have been associated with treatment response. Among genes implicated, those involved in dopamine signaling mediated by D2-class dopamine receptor, including *DRD2* itself and its molecular effectors, have been implicated as key genetic predictors of response to treatments. Studies have also reported that genetic variation in genes coding for proteins that cross-talk with DRD2 at the molecular level, such as *AKT1, GSK3B, Beta-catenin*, and *PPP2R2B* are associated with response to antipsychotics. In this review we discuss the relative contribution to antipsychotic drug responsiveness of candidate genes and GWAS identified genes encoding proteins involved in dopamine responses. We also suggest that in addition of these older players, a deeper investigation of new GWAS identified schizophrenia risk genes such as *FXR1* can provide new prospects that are not clearly engaged in dopamine function while being targeted by dopamine-associated signaling molecules. Overall, further examination of genes proximally or distally related to signaling mechanisms engaged by medications and associated with disease risk and/or treatment responsiveness may uncover an interface between genes involved in disease causation with those affecting disease remediation. Such a nexus would provide realistic targets for therapy and further the development of genetically personalized approaches for schizophrenia.

## Introduction

Whole genome association studies (GWAS) have identified several single nucleotide polymorphisms (SNPs) associated with enhanced risk for schizophrenia (1). These studies underscore the extraordinary polygenicity of schizophrenia and raises the hope that understanding the developmental causes of the disease may lead to new therapies (2, 3). Yet, most of these SNPs were not found in genes traditionally associated to the mechanism of action of existing antipsychotic drugs and it remains unclear whether it will be practically possible to intervene on pathways involved in disease causation.

Alternatively, several genetic factors have been shown to modulate the severity of schizophrenia symptoms and treatment responsiveness. Twins studies have demonstrated that, like pathophysiology and risk for schizophrenia, response to antipsychotic medication is a heritable trait (4, 5). Furthermore, reports have highlighted that schizophrenia risk, pathophysiology and response to treatments likely share a common genetic background (6). Based on such evidence, GWAS are currently considered a powerful tool to identify new molecular targets for antipsychotic treatments, while confirming the involvement of the already established ones. Genes and related proteins belonging to dopaminergic signaling have consistently been indicated as key elements for antipsychotics treatment responsiveness by both hypothesis- and data-driven pharmacogenetic studies.

The neurotransmitter dopamine is involved in the regulation of several cerebral functions including, reward, mood, sensory motor gating, affect and locomotor functions, learning and motivation (7, 8). Importantly, dopamine receptors, especially the D2 dopamine receptor (DRD2), are major pharmacological targets of all existing antipsychotic drugs (9–11) (Table 1). Several dopamine producing neuron populations exist in the adult brain. Cells from the nigrostriatal pathway project from the *substantia nigra pars compacta* to the caudate nucleus and the *putamen* in the striatum (12, 13). Dopamine neurons from the mesolimbic pathway (14) project from the ventral tegmental area to the ventral striatum, amygdala, and several cortical areas (e.g., pre-frontal cortex) expressing dopamine receptors (15). Dopamine neurons from the infundibular nucleus of the hypothalamus are also involved in the dopamine-mediated regulation of the pituitary gland (16). Among these neuronal networks, the mesolimbic pathway has received the most attention in the context of schizophrenia.

**Table 1:** List of main First-Generation (FGA) and Second-Generation (SGA) Antipsychotics with respective target Dopamine (DA) Receptors (D1–D5).

**Antipsychotic**		**Target DA receptor**				
		**DRD1**	**DRD2**	**DRD3**	**DRD4**	**DRD5**
FGA	Haloperidol	–	+++	++	+	–
	Chlorpromazine	+	+++	++	–	?
	Amisulpiride	–	++++	++++	–	?
	Perphenazine	?	++++	–	–	?
SGA	Aripiprazole	+	++++ PA	+++ PA	+ PA	–
	Clozapine	++	++	++	+++	+
	Olanzapine	+++	+++	+++	+++	+
	Lurasidone	?	+++	?	?	?
	Paliperidone	++	+++	+++	++	?
	Quetiapine	+	++	++	+	–
	Risperidone	+	+++	++	+++	+
	Ziprasidone	+++	+++	+++	+++	+
	Asenapine	++	++	+++	++	?
	Iloperidone	+	+++	+++	++	?

+ *Low Affinity*.

++ *Medium Affinity*.

+++ *High Affinity*.

++++ *Very High Affinity;—No Affinity; ? Uncertain data*.

*PA, Partial Agonist*.

Here, we will provide a brief overview of the biological regulation of dopamine and of the cellular mechanisms engaged by its receptors; for readers that would want a more complete description of the biology of dopamine receptor we would suggest more exhaustive reviews (7, 17, 18). We will then examine how molecular pathways involved in dopamine function intersect with genetic factors for antipsychotic drug responsiveness and, if applicable, disease causation. To evaluate this, two investigators independently conducted a systematic PubMed search (June 2018) for studies of antipsychotic medication responsiveness involving dopamine signaling cascade and related genetics. Combinations of the following keywords were used: Antipsychotics (APs), first-generation APs, second-generation APs, AP response, AP pharmaco-genetics, AP pharmaco-genomics, schizophrenia risk, schizophrenia genetics, schizophrenia genomics, schizophrenia polygenic risk score, dopamine, dopamine receptors, dopamine signaling. Finally, we will underscore how a newly discovered pathway may provide new avenues to investigate the genetic of antipsychotic drug responsiveness.

## Dopamine Homeostasis

**Figure 1:**
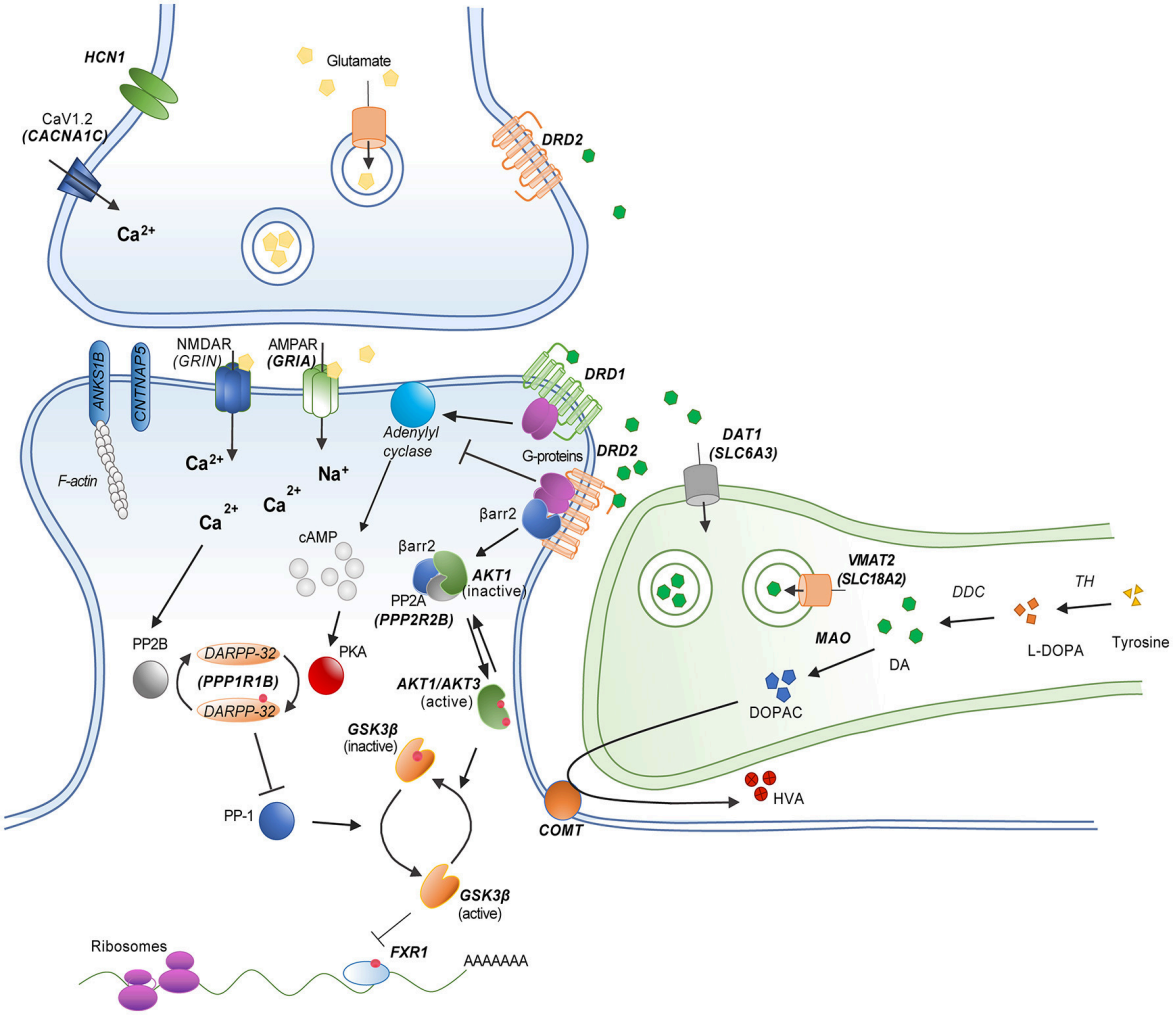
Schematic model of a striatal dopamine/glutamate synapse showing the functional relationship between different dopamine related gene products. Genes implicated in candidate gene studies and/or GWAS are indicated in bold. Red dots represent phosphorylation events.

During dopamine synthesis, the amino acid tyrosine undergoes hydroxylation to levodopa that is catalyzed by the enzyme tyrosine hydroxylase (TH) (Figure 1). This is followed by a decarboxylation of levodopa to dopamine by the enzyme dopa-decarboxylase (DDC) (19). In adult neurons, dopamine is loaded into synaptic vesicles by the vesicular monoamine transporter 2/solute carrier family 18 member A2 (VMAT2/SCL18A2) (20). Following synaptic release, dopamine can either be preferentially up-taken or degraded, depending upon neuronal circuit. The major transporter in charge of dopamine reuptake is the dopamine transporter/solute carrier family 6 member 3 (DAT1/SLC6A3) (21). This transporter is the key element in the mechanism removing extracellular dopamine in striatal structures. However, dopamine has also been shown to be reuptaken by other transporters such as the norepinephrine transporter/solute carrier family 6 member 2 (DAT/SLC6A2) in brain regions expressing low levels of the SLC6A3 gene product (22, 23). The major enzyme in charge of dopamine degradation is the Catechol-O-methyltransferase (COMT), which methylates dopamine into 3-metoxytyramine (3-MT). Degradation by COMT is the principal mechanism of dopamine clearance in the pre-frontal cortex. Dopamine can also be oxidized into 3,4-Dihydroxyphenyl-acetic acid (DOPAC) by the monoamine oxidases (MAOA and MAOB). The final step of dopamine metabolism involves the production of homovanillic acid (HVA) from 3-MT by monoamine oxidases or from DOPAC by COMT (24).

## Dopamine Receptors Signaling Mechanisms

Dopamine exerts its effects via the stimulation of five different G-protein coupled dopamine receptors (Figure 1). These receptors are divided in two groups based on their coupling and gene structures (17). D1-class dopamine receptors DRD1 and DRD5 are mostly coupled to G_α*s*_ G-proteins and encoded by genes that are devoid of introns. D2-class dopamine receptors DRD2, DRD3, and DRD4, are encoded by genes that comprise introns and are generally coupled to G_α*i*/*o*_ G-proteins. D1-class receptors mediate post-synaptic responses to dopamine. In contrast D2-class receptors can both mediate post-synaptic responses and act as presynaptic auto-receptors to limit dopamine synthesis and release (19). Of note, the *DRD2* gene encodes two splice variants of the receptor. The long isoform (D2L) is mostly expressed on post-synaptic neurons while the short isoform (D2S) is preferentially expressed by pre-synaptic dopamine neurons (25).

Activation of D1-class receptors results in an increased production of the second messenger cyclic Adenosine Monophosphate (cAMP) by class 3 adenylyl cyclases (ADCY) (26). Activation of D2-class receptor results in a reduction of cAMP by inhibiting this same mechanism (27). Major downstream effectors of dopamine receptors are cAMP-dependent protein kinases A (PKA) (7). PKA are holoenzymes comprised of a catalytic subunit and different regulatory subunits. Catalytic subunits are encoded by the genes *PRKACA, PRKACB*, and *PRKACG*. Regulatory subunits are encoded by the *PRKAR1A, PRKAR1B, PRKAR2A*, and *PRKAR2B* genes in humans. Regulation of PKA activity by dopamine receptors is involved in several cellular processes including, among others, the regulation of gene expression by transcription factors and the regulation of ionotropic receptors for various neurotransmitters. Among several targets of interest, the activity of cAMP response elements binding proteins family of leucine zipper transcription factors (i.e., CREB1) can be modulated by dopamine. Subunits of AMPA and NMDA ionotropic glutamate receptors (i.e., GRIN1, GRIA1, GRIA4) are also regulated by PKA downstream of dopamine receptors (7, 18). Finally, the protein phosphatase 1 regulatory subunit 1B (PPP1R1B/DARPP-32) has been shown to be regulated by dopamine and cAMP and to play a role in the balance of phosphorylation/dephosphorylation of several PKA substrates involved in dopamine receptor signaling and the integration of metabotropic (slow) and ionotropic (fast) neurotransmission (28, 29).

The signaling of dopamine receptors is not restricted to the regulation of cAMP production. Some receptors have been reported to have the possibility to couple to G_α*Q*_ G-proteins to regulate intracellular inositol and calcium signaling (30, 31). Furthermore, activation of G_βγ_ G-protein subunits by DRD2 results in neuronal hyperpolarization by regulating the activity of L and N-Type calcium channels (LTCC and NTCC) and G-protein gated inwardly rectifying potassium channels (e.g., GIRK2/KCNJ6) (7). Furthermore, DRD2 modulates neuronal function by acting on G-protein independent mechanisms. Following their activation, dopamine receptors are phosphorylated by G-protein receptor kinases (e.g., GRK2, GRK6) (32). This leads to the recruitment of beta-arrestins (ARBB1 and ARBB2), which inactivate G-protein coupling, stimulate receptor internalization and mediate additional cell signaling functions (33, 34). In the case DRD2, the recruitment of ARBB2 results in the formation of a protein complex that favors the inactivation of protein kinases from the Akt family (AKT1, AKT2, AKT3) by protein phosphatase 2 holoenzymes (i.e., PPP2R2B, PPP2CA, PPP2CB). The inactivation of AKT kinases downstream of DRD2 releases the inhibition of glycogen kinase 3 family proteins (GSK3A, GSK3B) thus increasing their activity (35, 36). The nature of the GSK3 substrates involved in DRD2 signaling is still unclear. One frequently investigated potential target is the canonical wingless (WNT) signaling transcription factor beta-catenin (CTNNB1) (37). However, beta-catenin does not appear to be a major determinant of antipsychotic drug action downstream of DRD2 in mice (38). The RNA binding protein fragile-X-mental-retardation-autosomal-homolog-1 (FXR1) is a recently identified GSK3B substrate (39) that has been shown to modulate ionotropic glutamate receptor functions *in vivo* (40, 41). It is thus possible that FXR1 may contribute to the regulation of neuronal functions by GSK3-mediated DRD2 signaling (42).

## Candidate Gene Studies

Candidate gene studies have often been focused on the role of dopamine receptors and related genes (namely *DRD1, DRD2, DRD3, DRD4*, and *DRD5*) as modulators of antipsychotic drug responsiveness and schizophrenia symptoms. In particular, genetics studies on schizophrenia endo-phenotypes, i.e., behavioral and/or neuroimaging phenotypes associated with the disorder, have suggested that variations in genes coding for this cluster of receptors, especially those coding for DRD2, DRD3, and DRD4, are associated with cognitive deficits and related brain pre-frontal cortex malfunction, which are typical phenotypes of schizophrenia. Candidate gene investigations have identified allelic variation in SNPs of functional relevance to D2, D3, and D4 receptors to be associated with response to antipsychotic medication (Table 2).

**Table 2:** Genes and corresponding mutation implicated in antipsychotic response/resistance by Candidate gene studies or GWAS.

	**Gene**	**Mutation**	**Biological function/effect**	**Study**	**Pharmacogenetic effect**
Candidate genes	*DRD2*	rs1800497 or Taq1A	Missense, Glu-to-Lys substitution at position 713; affects *DRD2* mRNA stability and expression along with striatal DRD2 density and binding	(43)	Nemonapride response
				(44)	Haloperidol response
				(45)	Risperidone response
				(46)	Aripiprazole response
		rs1801028	Ser-to-Cys substitution at position 311; affects the physiology and function of DRD2 receptor	(47)	Risperidone response
		rs1079597 or Taq1B	B1 allele associated with reduced DRD2 density in striatum	(48)	B2 allele associated with response to Clozapine
		rs1799732 or −141C Ins/Del	Deletion (vs. insertion) of cytosine at position-−141, located in the 5′ promoter region	(49)	Chlorpromazine response
		rs2514218	Genome Wide Association with SCZ	(50)	Risperidone and Aripiprazole response in first episode psychosis
	*DRD3*	rs6280	Serine to glycine substitution at amino acid position 9; associated with altered DRD3 dopamine affinity and density in some brain areas	(51)	Clozapine response
				(52)	Positive symptom response to Olanzapine
				(53)	Aripiprazole response
	*DRD4*	rs4646984 or 48bp-VNTR	48 bp Variable Number Tandem Repeat (48.bp VNTR) falling within the third exon of the gene	(54)	Response to multiple neuroleptics
				(55)	Clozapine response as compared to other APs
				(56)	Clozapine response
				(57)	Response to different APs
	*COMT*	rs4680 or Val108Met	Met/Met subjects have lower enzyme activity (hence, reduced prefrontal dopamine clearance) as compared to Val/Val individuals	(58)	Met/Met associated with reduced response to FGA
				(59)	Val/Val associated with reduced response of Negative Symptoms to Olanzapine
				(60)	Met/Met associated with better response of Cognitive Symptoms to Clozapine
				(61, 62)	Interaction with *NOTCH4* SNP2 polymorphism on response to FGA
				(62)	Response to multuiple-AP treatment
				(63)	Risperidone response
				(64)	Risperidone response
	*SLC6A3*	3'VNTR	SLC6A3 expression levels	(65)	AP resistance
GWAS genes	Unknown	rs17390445	Genome Wide Association with SCZ; located on Chromosome 4 in intergenic position; unknown function	(66)	Ziprasidone response
	*ANKS1B*	rs7968606	Unknowkn	(66)	Olanzapine response
	*CNTNAP5*	rs17727261	Unknown	(66)	Risperidone response
	*AKT1*	rs1130233	*AKT1* mRNA expression levels along with level of phosphorylation of the AKT1 kinase	(67)	Interaction with DRD2 rs1076560 on Olanzapine response

*Putative biological impacts along with pharmacogenetics results are also reported*.

Genetic studies on the role of *DRD2* genetic variation in the pathophysiology and risk for schizophrenia and response to antipsychotics have been motivated by the evidence that DRD2 antagonism is a key mechanism of action of antipsychotic agents to the extent that occupancy of this receptor subtype is correlated with antipsychotic potency (Table 1). The *DRD2* locus is the only dopamine receptor gene for which common variants have been associated to schizophrenia risk by GWAS (1). Candidate gene studies on functional polymorphisms of *DRD2* gene, involving SNPs that affect *DRD2* gene expression and/or *DRD2* Long/Short transcription isoform ratio, along with DRD2 membrane density, have also pointed to a role of such genetic variation in schizophrenia. For example, rs1800497 (also known as Taq1A) is a missense variant determining a Glu-to-Lys substitution at position 713 of *DRD2* gene and associated with the gene mRNA stability and expression (68), striatal dopamine receptor density (69), and modification in DRD2 binding in human striatum (70). Interestingly, rs1800497 has been associated with performance in Working Memory, a prototypical endophenotypes of schizophrenia, mainly subtended by pre-frontal cortex activity (71). Similarly, rs1801028, a SNP occurring in *DRD2* gene and determining a Ser-to-Cys substitution at position 311 of *DRD2* gene (Ser311Cys) has been shown to alter the physiology and function of DRD2 receptor (72) and has consistently been associated with risk for schizophrenia in a number of studies since 1994 (73)—for a meta-analysis see: (74). Another example has been provided by rs1076560, a SNP located in intron 6 of the *DRD2* gene, whose T allele has been reported to shift the splicing of *DRD2* transcript from D2S to D2L isoforms (75). Rs1076560 has been associated with risk for schizophrenia (76), behavior and brain activity during cognitive and emotion processing in healthy controls and patients with schizophrenia (77), with efficiency of pre-fronto-striatal activity during Working Memory (78) and levels of striatal dopamine (79).

Since DRD2 neuronal signaling is mediated by a number of partner molecular elements downstream of the receptor, including proteins belonging to cAMP-dependent and cAMP-independent pathways, such as AKT1, GSK3B, PPP2R2B, and CTNNB1, not surprisingly also genes coding for such proteins have been associated with phenotypes that are relevant to schizophrenia pathology. For example, rs1130233, a *cis*-eQTL of *AKT1* gene that interacts with rs1076560 in affecting pre-frontal *AKT1* mRNA expression levels along with level of phosphorylation of the AKT1 kinase target GSK3beta, interacts with rs1076560 also on cingulate cortex activity during attentional control, another cognitive function typically impaired by schizophrenia (67). Similarly, rs12630592 (80) and rs609412 (81), two eQTLs of *GSK3beta* and *PPP2R2B* gene, respectively, have been associated with cognitive performance and related brain activity critically implicated in the disorder.

Functional variation of *DRD2* gene has also been studied as predictor of antipsychotic response, (for a review see: (82). In general, dopamine receptors variants associated with reduced expression of the receptor protein or altered functioning were also associated with poorer response to antipsychotic drugs, confirming that these receptors mediate antipsychotic activity. Rs1800497 has been associated with response to nemonapride (43), haloperidol (44), risperidone (45), and clozapine (48). Similarly, rs1801028 has been associated with response to risperidone (47), while interaction between rs1076560 and rs1130233 has been implicated in response to olanzapine as measured by variation in Positive and Negative Symptoms Scale total scores after 56 days of stable dosage treatment (67). Other SNPs in *DRD2* have been associated with antipsychotics response, including rs1079597, also known as Taq1B, whose B1 allele has been associated with reduced DRD2 protein density in the striatum, and B2 allele has been implicated in response to clozapine (48). Further mutations have been associated with clinical response to chlorpromazine (49) and aripiprazole (46).

*DRD3* has systematically been studied by pharmacogenetics since many antipsychotic drugs show high affinity for the D3 dopamine receptor (83). A missense polymorphism in exon 1 of *DRD3* leading to a serine to glycine substitution at amino acid position 9 (rs6280) has been associated with altered DRD3 dopamine affinity and density in some brain areas (84) along with response to first-generation antipsychotics and lack of response to clozapine (51). Some studies (52) found rs6280 to be associated with greater acute positive symptom remission after olanzapine treatment. The same effects were observed for different SNPs in linkage disequilibrium with rs6280. Further studies have also implicated rs6280 in response to the second-generation antipsychotic aripiprazole (53).

Evidence has also established a correlation between response to antipsychotics and functional genetic variation of the *DRD4* gene. In particular, since DRD4 is targeted by clozapine, the most effective antipsychotic drug currently available (85), studies have particularly focused on the effect of genetic variability of corresponding gene on response to such a drug. One of the numerous polymorphisms occurring in *DRD4* gene (namely, rs4646984), is a 48 bp Variable Number Tandem Repeat (48.bp VNTR) falling within the third exon of the gene and resulting in a different length of the third cytoplasmatic loop of DRD4 protein. Importantly, the longer repeat version of the protein has been associated with reduced clozapine binding, thus suggesting the 48 bp VNTR to be an interesting candidate polymorphism to study as predictor of clinical response to this antipsychotic. However, while some studies have reported an effect of rs4646984 on responsiveness to various antipsychotics including clozapine (54–57) several other studies also reported no associations, as reviewed in Zhang and Malhotra (83).

Along with genes coding for dopamine receptors, other genes belonging to the dopaminergic system have also been implicated in pathophysiology and risk for schizophrenia, hence becoming candidate genes for as regulators of response to antipsychotics (Table 2). The *COMT* and *SLC6A3* genes, have been particularly relevant within this framework.

Studies have consistently demonstrated that a common polymorphism in *COMT*, rs4680 or Val108Met, is responsible for a functional variation in enzymatic activity with the Met/Met condition characterized by lower activity (hence, reduced pre-frontal dopamine clearance) as compared to the Val/Val condition. A large number of studies have investigated the genetic association between rs4680 and the diagnosis of schizophrenia with mixed results—for a meta-analysis see: (86). In line with the role of COMT in dopamine turnover, pharmacogenetic investigations have reported an association between rs4680 allelic variations and the clinical response to antipsychotics acting on dopamine receptors. An early study found that individuals with schizophrenia carrying a met/met genotype were less responsive to first-generation antipsychotics (58). The val/val genotype has also been associated with reduced response of negative symptoms to olanzapine (59) while the met/met genotype has been associated with increased response of cognitive symptoms to clozapine (60). A number of additional studies, then, confirmed the impact of the COMT Val158Met polymorphism on the efficacy of first-generation (61, 62) and second-generation antipsychotics (63, 64, 87).

Studies have also implicated *SLC6A3*, coding for the dopamine transporter, in schizophrenia phenotypes and response to medication. A functional Variable Number of Repeats polymorphism at 3' end of this gene (3'-VNTR) has been described (88, 89). Alleles of this polymorphism range from 3 to 11 repeats, with the 9- and 10-repeat alleles by far the most common (88, 89). As compared with the 9-repeat allele, the 10-repeat allele has been associated with increased *SLC6A3* gene expression both *in vitro* (90, 91) and in human striatum (92). Furthermore, studies have reported that the 10-repeat allele is associated with more focused cortical activity during memory and attention, two critical endophenotypes of schizophrenia (93–98). Evidence has also documented an interaction between *DRD2* rs1076560 and *SLC6A3* 3'-VNTR on further imaging phenotypes associated with the disorder, such as pre-fronto-striatal activity and volume in humans (77).

Although not directly targeted by antipsychotic medications, the dopamine transporter, may influence dopamine-signaling intensity by virtue of its function of dopamine clearance at the synaptic level and contribute to treatment outcome. Consistently, a number of studies have reported association between polymorphisms in the gene coding for this transporter, including 3'-VNTR and level of response to first and second-generation antipsychotics (65, 99).

## Genome Wide Association Studies

GWAS have allowed for detection of genetic variants associated with a specific disease without any *a priori* hypothesis on its pathophysiology (hypothesis-free or data-driven studies). Such an approach has confirmed the polygenic nature of risk for schizophrenia since it has identified more than one hundred genetic variants associated with the disorder at genome wide statistical level of significance (i.e., *p* < 10^−8^) (1). Furthermore, studies attempting at collapsing GWAS multigenic risk in so called Polygenic Risk Scores (PRSs) have probed these scores to predict behavioral and neuroimaging phenotypes of schizophrenia (81). Nevertheless, since GWAS and PRSs do not provide any specific insights into the biological role of genetic variation associated with a disease, different approaches have been developed in order to dissect risk into biologically meaningful pathways and probe the impact of pathway-specific risk variants and PRSs onto pathophysiology of schizophrenia and related phenotypes (81).

Several genes, including *DRD2*, have been associated with schizophrenia by GWAS, even though, most findings have implicated different variants from those detected by candidate gene approaches (1). Nonetheless, as for candidate genes investigation, risk variants identified by GWAS have been implicated in response to antipsychotic medication, confirming the hypothesis that schizophrenia pathophysiology and response to treatment share genetic bases that are partially involving dopamine signaling. A prototypical example is provided by rs2514218, a SNP in *DRD2* gene that was associated with schizophrenia in a large GWAS published by the Psychiatric Genomics Consortium (1). Zhang and collaborators (50) examined whether genotype at this SNP could predict response to 12 weeks of risperidone or aripiprazole treatment in a cohort of patients with first episode of psychosis and found that homozygotes for the risk (C) allele at this SNP had significantly greater reduction in positive symptoms after treatment with either risperidone or aripiprazole as compared to the T allele carriers.

From a pharmacogenomics perspective, a pivotal role in the study of antipsychotics response has been played by the Clinical Antipsychotics Trials of Intervention Effectiveness (CATIE) (100), a multicenter research project promoted by the USA National Institute of Health and investigating the effectiveness of first and second-generation antipsychotics. Within the CATIE, a number of GWAS have been conducted on both treatment response and adverse reaction (Table 2).

The first GWAS (66) found a SNP (rs17390445) located on Chromosome 4 in an intergenic position, to be associated with response of schizophrenia positive symptoms to the second-generation antipsychotic ziprasidone, while another SNP in the same intergenic region approached, but did not reach, genome wide significance. Both rs17390445 and this second SNP functions remain unknown, but involvement in dopamine signaling cannot be excluded. Interestingly, in a different study, the same group looked at neurocognition as an outcome of antipsychotics response (101) and found a weak association with SNPs in *DRD2* gene. This same study identified SNPs associated with olanzapine and risperidone response in Ankyrin Repeat and Sterile Alpha Motif Domain-Containing Protein 1B (*ANKS1B*) and in the Contactin-Associated protein-Like 5 genes (*CNTNAP5*) which, are not directly connected with dopamine signaling, but are involved in interneuron communication.

GWAS were also used to explore the clinical response to new antipsychotics partially exerting their pharmacological action by blocking dopamine transmission. For example, two of these studies (102, 103) investigated genome wide association with response to Iloperidone, an antagonist of D2 and D3 dopamine receptors also blocking noradrenergic and serotonergic neuronal signaling. None of the SNPs reaching genome wide significance were directly involved in dopaminergic system, even though one of the genes associated, *GRIA4*, codes for AMPA 4 glutamate receptor, that may impact on dopamine neurotransmission by mediation of glutamate signaling. In fact, evidence suggests that aberrant glutamatergic function may alter dopamine system function in psychotic disorders (104, 105) and dopamine exert several of its biological actions by modulating ionotropic AMPA and NMDA glutamate receptor functions (7, 29). Furthermore, dopamine D2/D3 receptor availability is linked to the severity of psychotic symptoms induced by glutamatergic antagonism (106) suggesting that factors influencing glutamate signaling may contribute to dopamine dysregulation and symptoms or response to dopamine-mediated treatments (107). Interestingly, a study (108) looking at drugs targeting proteins encoded by genes GWAS associated with schizophrenia, found that antipsychotics are the only medication surviving enrichment procedures and that, two of the four genes associated with antipsychotic response were either directly implicated in glutamatergic signaling (namely, *GRIN2A*) or indirectly related to dopaminergic system (i.e., *AKT3*). The remaining two genes, *CACNA1C* and *HCN1*, respectively coding for LTCC Ca_v_ 1.2 and Hyperpolarization-activated cyclic nucleotide-gated (HCN) channels, have also been implicated in dopamine signaling modulation (109, 110).

GWAS-based pharmacogenomics studies on antipsychotic response have also used PRS strategies in order to explore the cumulative role of genetic risk on pharmacological effects. For example, one study (111) showed a PRS based on schizophrenia-associated SNPs reported by the Psychiatric Genomics Consortium (1) to predict clozapine response, in that clozapine responders had higher PRS as compared to non-responders. Furthermore, a study using a multistage GWAS-PRS-Pathway Analysis approach (6) to detect genetic variation associated with response to the second-generation antipsychotic lurasidone, suggested associated variants to belong to functional categories of relevance to neuronal transmission and being, at least partially, involved in dopamine signaling by impacting on glutamate an serotonin systems modulation.

## An Interesting New Candidate Gene, the Fragile X Autosomal HOMOLOG 1

At first glance, most schizophrenia risk SNPs identified by GWAS are not associated to genes encoding products generally related to dopamine transmission or cellular responses to antipsychotic drugs (1). However, some of these loci may still encode proteins regulated by therapeutic agents. Investigating such genes in the context of known mechanisms of antipsychotic actions may thus be a fruitful avenue for future studies.

For example, the RNA binding protein FXR1 encode on chromosome 3q26.33 represents an interesting new candidate among the genetic risks factors for schizophrenia identified by GWAS (1). FXR1 belongs to the fragile X proteins family, which comprises three RNA-binding proteins members—Fragile X mental retardation protein (FMR1/FMRP), and two Fragile X related proteins—FXR1 and FXR2 (112, 113). All three proteins play a role in regulating the development of several tissues including the brain. Furthermore, these proteins are also implicated in the regulation of neuronal functions (114–116).

The Fragile X mental retardation syndrome is a primary monogenic cause of autistic spectrum disorders (ASDs) and is caused by mutations in FMR1 gene, which is highly expressed in neurons and plays a crucial role in regulating synaptic plasticity (117–119). Both FXR1 and FXR2 have also been recently shown to participate in synaptic plasticity (40, 41, 120). Proteins from this family comprise homologous amino-terminal regions containing a tandem Tudor domain which can mediate binding to methylated lysine on histones (121). The amino-terminal region of these proteins also includes a nuclear localization and export signals (NLS and NES) that mediate shuttling between nucleus and cytoplasm (122). The medial regions of FMR1, FXR1, and FXR2 comprise RNA-binding KH domain and RGG box that allow RNA-binding (123). Finally, the three proteins sequences are most divergent at the carboxyl-terminal domain, which seem to functionally distinguish members of the family (124). All three fragile X family proteins participate to the regulation of mRNA translation, degradation, and are associated with ribosomes (116, 123, 125).

FMR1 is by far the most studied of these three proteins. Interestingly, an exome study of rare genetic variants in schizophrenia identified an enrichment for gene encoding mRNA that are associated to FMR1 (126). Furthermore, a postmortem study has shown altered protein levels of FMR1 targets in the frontal cortex of subjects with schizophrenia or bipolar disorder (127). However, SNPs in *FMR1* are not associated to schizophrenia as defined by a GWAS studies of common genetic risk variants (1). In contrast to FMR1, FXR1 came out of the shadow after its identification as one of the top 30 potential genetic risk factor for schizophrenia (1). For a long time FXR1 was considered either a protein functionally redundant to FMR1 (125) or as its muscle specific homolog (115). Both FXR1 and FMR1 have been shown to interact together but can also have mutually exclusive functions and cellular localization (128). It would thus be premature to conclude that changes in FMR1 targets identified in schizophrenia results from altered FXR1 functions since these two proteins are not biologically equivalent.

**Figure 2:**
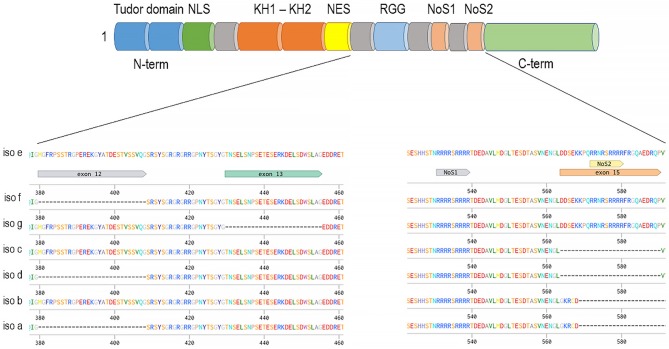
Fxr1 gene (mouse gene depicted as an example) alternatively spliced mRNAs and common domain structure on example of the longest isoform—e. The presence of exon 15 provides an additional nucleolar targeting signal.

The *FXR1* gene encodes seven major alternatively spliced mRNA variants (Figure 2, mouse gene used as an example), three of which are expressed specifically in muscles and testis (115, 129). Characterization of the schizophrenia risk allele in the *FXR1* locus (rs34796896) has shown it to be in linkage disequilibrium with splicing quantitative trait loci (sQTL) SNP (rs1805564) identified in *postmortem* human dorsolateral pre-frontal cortex (DLPFC). Altered splicing in the locus of exon 15 is associated and functionally changed in presence of the rs34796896 schizophrenia risk allele (130). The expression of exon 15 containing isoforms of *FXR1* has only been demonstrated in muscle and testicular tissues by means of western blot. However, the presence of exon 15 RNAs has been detected by sequencing in other tissues, thus suggesting that minor amounts of the protein carrying this exon could be expressed outside of the muscle and testis (130). Similar non-canonical expression of intronic sequence possessing novel isoform of FXR1 in CD4+ T cells was also shown by means of mass spectrometry as a confirmation of RNA-sequencing data (131).

Exon 15 provides an additional nucleolar-targeting signal (NoS2), which is necessary for efficient shuttling between cytoplasm and nucleoli. This shuttling was demonstrated in Cos-7 cell line for the isoform *e* of FXR1 as well as for FXR2 but not for FXR1 isoform *d* or FMR1 (132). Lately, nucleolar localization was also demonstrated for FMRP in Hela cells where FMRP expression is high (133). Interestingly, unlike other family members that exhibit a similar cytoplasmic localization between fetal and adult tissues, FXR1 has been reported to have nuclear localization only during brain and muscle development (114, 115). Thus, it is possible that alteration in the nuclear or nucleolar localization of FXR1, regulated by the NoS2 of exon 15, may contribute to increase the risk to develop schizophrenia.

FXR1 may also contribute to DRD2 and GSK3β mediated signaling (42). Indeed, GSK3β, which is activated following DRD2 stimulation and inactivated by several antipsychotics (134), is able to directly phosphorylate Fxr1 and regulate its expression in a negative manner (39). Furthermore, chronic treatment with lithium and valproate resulting in an inhibition of GSK3β activity also increased Fxr1 expression in the mouse striatum and pre-frontal cortex (39). Interestingly, GSK3β and FXR1 are not affected by chronic lithium or valproate treatment in mice lacking ARBB2 (39). Furthermore, lithium also fails to engage AKT-GSK3 mediated signaling in mice lacking either ARBB2 of DRD2 (135, 136), which suggests an engagement of the DRD2-ARBB2-AKT-GSK3 pathway in the regulation of Fxr1 levels.

A functional interaction has been reported between SNPs affecting the relative expression of the *FXR1* (rs496250) and *GSK3B* (rs12630592) genes (39). In healthy humans, this interaction has been shown to affect amygdala response to emotional faces, as measured by functional Magnetic Resonance Imaging (fMRI). The same interaction was observed on measures of trait Emotional Stability as conceived within the Big Five Model of Personality (39). Finally, interaction between these functional SNPs has been replicated and showed to affect symptoms severity and, putatively, treatment responsiveness in bipolar patients (137). Evidence for a contribution of *FXR1* to dopamine signaling and responsiveness to psychiatric disorders remains preliminary. However, the existing observations would support that *FXR1* is one example of a gene for which further investigation of contribution to schizophrenia, DRD2 signaling, and antipsychotics drug responsiveness is warranted. Further examination of other GWAS identified loci may allow finding additional candidate genes of interest with more distal relations to dopamine neurotransmission.

## Conclusion

Genetic investigations of risk factors for schizophrenia and determinants of drug responsiveness revealed a very multi-genic landscape for both indications. This suggests that schizophrenia arises from a combination of multiple genetic and socio-environmental hits occurring during development. This is also in line with the variable profile of drug responsiveness observed at the clinical level. This picture can be discouraging as several risk factors may only participate to disease at a pre-onset stage or, contribute to ubiquitous functions across different organs, thus preventing their practical or ethical use as therapeutic targets. Nonetheless, the overlap of schizophrenia risk and genes affecting responsiveness to existing drugs also points toward a nexus of biological mechanisms engaged by medication and those contributing to disease etiology. This supports the idea that disease remediation can intersect with disease causation and help compensate for developmental insults, even during adulthood. The prevalence of genes involved in dopamine transmission among those associated with treatment responsiveness is flagrant. However, dopamine is an “old player” in schizophrenia therapy and the prevalence of dopamine-associated genes probably results from a bias induced by the mechanism of action of therapeutic agents. The further examination of genes that are more distal to dopamine signaling and are nonetheless associated with schizophrenia risk and drug responsiveness may provide a new roster of “prospects” that can be realistically targeted for therapies whether alone, or in conjunction with older dopamine-targeting therapeutics. Furthermore, the integrated investigation of new and old variants associated both to genetic risk and treatment responsiveness can provides the bases for the development of personalized treatment protocols for schizophrenia.

## Author Contributions

All authors wrote the manuscript. JB also provided funds for publication.

### Conflict of Interest Statement

The authors declare that the research was conducted in the absence of any commercial or financial relationships that could be construed as a potential conflict of interest.
